# Gadolinium Spin Decoherence Mechanisms at High Magnetic Fields

**DOI:** 10.1021/acs.jpclett.3c01847

**Published:** 2023-11-17

**Authors:** C. Blake Wilson, Mian Qi, Songi Han, Mark S. Sherwin

**Affiliations:** † Laboratory of Chemical Physics, National Institute of Diabetes and Digestive and Kidney Diseases, National Institutes of Health, Bethesda, Maryland 20892, United States; ‡ Faculty of Chemistry and Center for Molecular Materials, Bielefeld University, 33615 Bielefeld, Germany; § Department of Chemistry and Biochemistry, University of California, Santa Barbara, Santa Barbara, California 93106, United States; ∥ Department of Chemical Engineering, University of California, Santa Barbara, Santa Barbara, California 93106, United States; ⊥ Institute for Terahertz Science and Technology, University of California, Santa Barbara, Santa Barbara, California 93106, United States; # Department of Physics, University of California, Santa Barbara, Santa Barbara, California 93106, United States

## Abstract

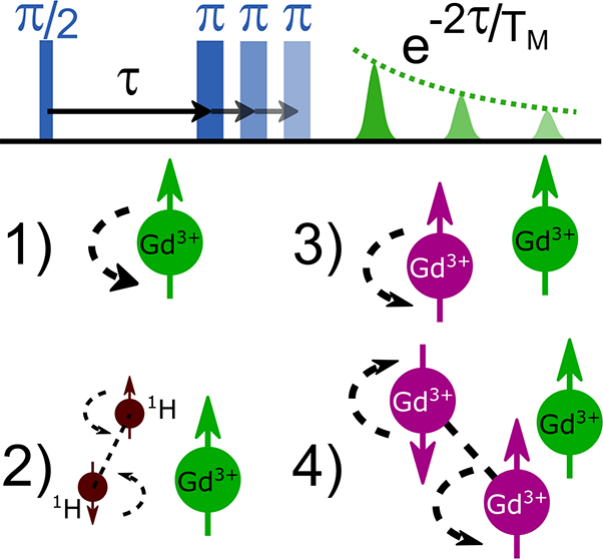

Favorable relaxation processes, high-field spectral properties, and biological compatibility have made spin-7/2 Gd^3+^-based spin labels an increasingly popular choice for protein structure studies using high-field electron paramagnetic resonance. However, high-field relaxation and decoherence in ensembles of half-integer high-spin systems, such as Gd^3+^, remain poorly understood. We report spin–lattice (*T*_1_) and phase memory (*T*_M_) relaxation times at 8.6 T (240 GHz), and we present the first comprehensive model of high-field, high-spin decoherence accounting for both the electron spin concentration and temperature. The model includes four principal mechanisms driving decoherence: energy-conserving electron spin flip-flops, direct “*T*_1_” spin–lattice relaxation-driven electron spin flip processes, indirect *T*_1_-driven flips of nearby electron spins, and nuclear spin flip-flops. Mechanistic insight into decoherence can inform the design of experiments making use of Gd^3+^ as spin probes or relaxivity agents and can be used to measure local average interspin distances as long as 17 nm.

Pulsed electron paramagnetic resonance (EPR) spectroscopy is a technique with broad applications in biochemistry, physics, and material science.^1−3^ Small molecules containing unpaired electrons in the form of organic radicals or metal ions can be site-specifically embedded into larger systems to act as sensitive reporters of local structure and dynamics.^3−5^ Pulsed dipolar spectroscopy techniques together with site-directed spin labeling are routinely used to measure pairwise distances between specific locations of proteins and other biomolecules^6^ to probe nanometer-scale structure. Additionally, molecules with unpaired electrons have been designed with promising potential applications as “molecular spin qubits” for quantum information science.^7−11^ All of these applications rely on coherent spin manipulations and are therefore ultimately limited by the electron spin coherence lifetime. Understanding and quantifying the particular physical processes driving spin coherence decay, also termed decoherence, dephasing, or transverse relaxation, are therefore of great importance for many magnetic resonance studies.

Decoherence mechanisms are especially poorly understood in high-spin systems. Molecules containing high-spin Gd^3+^ ions are of particular interest for pulsed dipolar EPR spectroscopy at a high magnetic field.^12−15^ Gd^3+^ ions have a spin-7/2 ground state with seven unpaired electrons in a half-filled 4f shell. Molecules containing Gd^3+^ ions typically have a relatively small zero-field splitting (ZFS) between 0.2 and 2 GHz^16^ and, hence, a narrow central *m* = −1/2 → *m* = 1/2 transition. This Kramers doublet is affected only by ZFS to second order in the perturbation theory, leading to a strong, narrow EPR central transition. At high magnetic fields *B*_0_, this central transition narrows as 1/*B*_0_, leading to field-dependent improvements in sensitivity and resolution.^12,17,18^ Long-range interactions between Gd^3+^ centers are stronger than between spin-1/2 centers, because Gd^3+^ possesses a large magnetic moment of 7 times that of a spin-1/2 system. At high magnetic fields and cryogenic temperatures, the spin–lattice relaxation characterized by the time constant *T*_1_ and the spin decoherence time *T*_M_ are both longer than those for most high-spin metal ions. Interestingly, *T*_M_ is typically observed to be only a factor of 5–10 shorter than *T*_1_ at high magnetic fields,^12,13^ in marked contrast to conventional spin-1/2 organic radicals, where *T*_M_ is typically 2–4 orders of magnitude shorter than *T*_1_.^19^

To study the mechanisms driving decoherence, with particular focus on quantifying the role of spin–spin coupling, we performed electron *T*_M_ and *T*_1_ measurements as a function of the temperature and electron spin concentration under conditions relevant for Gd^3+^ pulsed EPR spectroscopy applications.^20−22^ Measurements were carried out in frozen aqueous solutions containing Gd^3+^ chelates at 8.6 T, yielding an electron Larmor frequency ω_L_/2π = *g*μ_B_*B*_0_/ℏ = 240 GHz for the central *m* = −1/2 → *m* = +1/2 transition, where *g* = 1.992 is the isotropic Gd^3+^*g* factor, μ_B_ is the Bohr magneton, and *B*_0_ is the external magnetic field. Pulsed EPR experiments were performed using a home-built EPR spectrometer described elsewhere^23,24^ using a 55 mW solid-state microwave source (Virginia Diodes, Inc.), which can produce π pulses of approximately 275 ns. *T*_M_ was measured with a two-pulse electron spin echo decay experiment (Figure 1), consisting of a *P*_1_–τ–*P*_2_–τ–echo pulse sequence, where the echo was recorded as a function of the interpulse delay τ. Echo decay curves were well-described by an exponential and did not depend upon the pulse lengths used, indicating that instantaneous spectral diffusion was not significant (see Figure S4 of the Supporting Information). *T*_1_ was measured with a saturation recovery experiment, where a 300 μs pulse was used to saturate the EPR transition of the sample under investigation, and the recovered EPR was signal-readout after a variable recovery delay *T* with a two-pulse spin echo (Figure 1). A long pulse was used to ensure that the transition was adequately saturated by our low-power microwave source.

**Figure 1:**
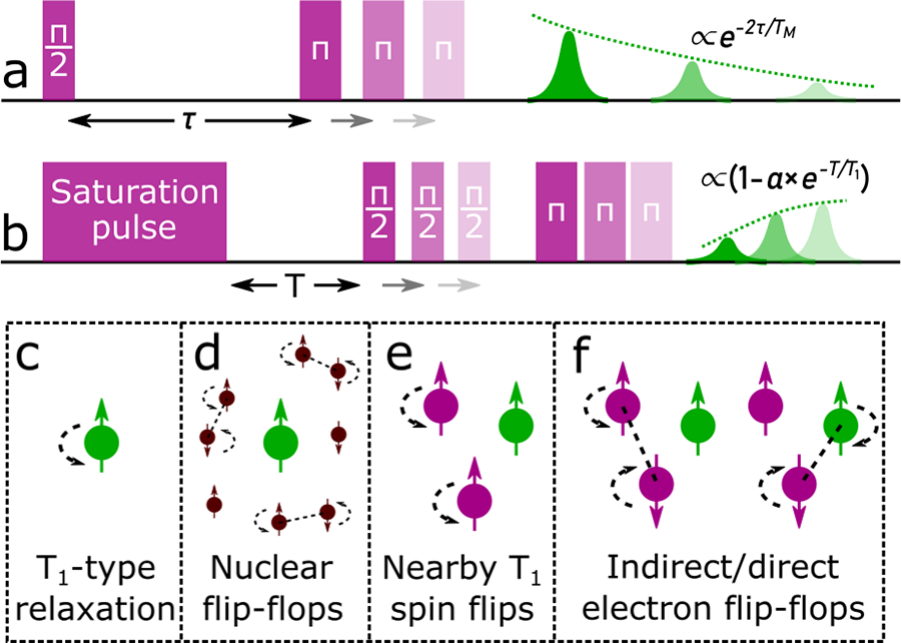
(a) Two-pulse electron spin echo decay decay pulse sequence used to measure the electron phase memory time *T*_M_. τ is varied, and the echo amplitude is recorded. (b) Saturation recovery pulse sequence used to measure spin–lattice relaxation time *T*_1_. The echo is recorded as the delay between the saturation pulse and the echo sequence *T* is increased, while keeping τ fixed. (c–f) Mechanisms driving decoherence, with “A” spins shown in green and “B” spins shown in purple. (c) Direct *T*_1_ processes. (d) Nuclear spin flip-flop mechanism. (e) Neighboring spin *T*_1_-induced spin flip mechanism. (f) Direct and indirect electron spin flip-flop mechanism.

Two Gd^3+^ complexes were studied, Gd-DOTA and iodo-(Gd-PyMTA) (Gd-PyMTA). Gd-DOTA is a commercially available magnetic resonance imaging (MRI) contrast agent with a small 0.7 GHz axial ZFS,^16^ while Gd-PyMTA is a pyridine-based tetracarboxylate ligand structure to be used as a transition-metal- or lanthanide-based spin probe with a ZFS of 1.2 GHz.^16^ Both Gd-DOTA and Gd-PyMTA can be functionalized as spin labels for biomolecular structure studies. Field-swept echo-detected spectra acquired around the central *m* = −1/2 → *m* = +1/2 transition as a function of the temperature (see Figures S2 and S3 of the Supporting Information) show that, for the outside of the central transition, the echo amplitude was <5% of the peak signal, indicating that only the central transition contributes significantly to the echo detected on resonance.

Figure 2 shows the electron spin–lattice rate 1/*T*_1_ measured on the central *m* = −1/2 → *m* = +1/2 transition between 10 and 50 K. For both Gd-DOTA and Gd-PyMTA, 1/*T*_1_ was observed to follow a power-law temperature dependence, consistent with *T*_1_ dominated by a direct phonon relaxation process.^25^*T*_1_ was not observed to change as the electron spin concentration was increased from 50 to 500 μM.

**Figure 2:**
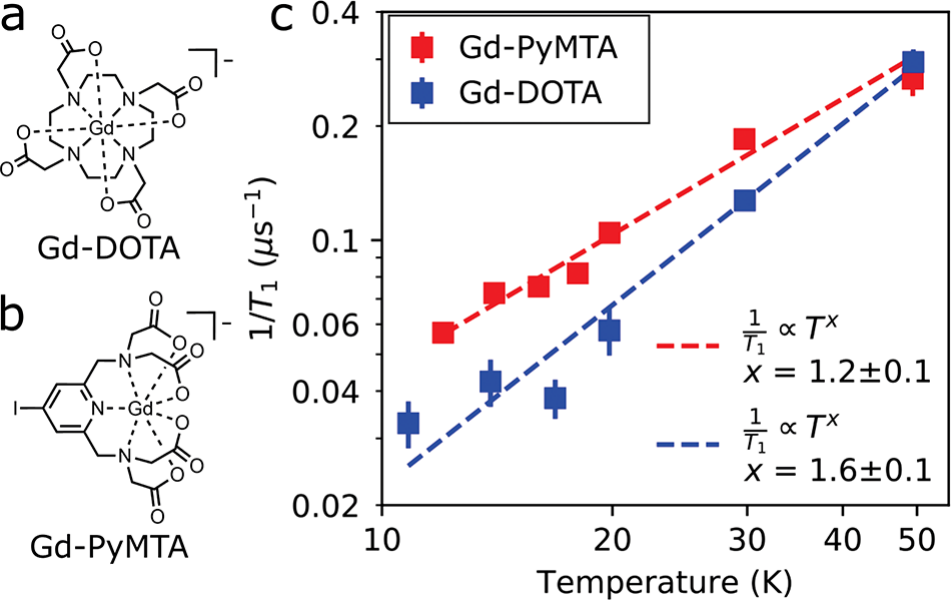
(a) Structure of Gd-DOTA. (b) Structure of Gd-PyMTA. (c) Inverse spin–lattice relaxation time 1/*T*_1_ at 8.6 T/ω_L_ = 240 GHz as a function of the temperature measured in frozen 60:40 deuterated glycerol/D_2_O for Gd-DOTA and Gd-PyMTA at a concentration of 500 μM. Dashed lines indicate power-law fits, consistent with *T*_1_ being driven by a direct phonon relaxation process.

Panels a and b of Figure 3 show the results of electron spin echo decay experiments performed on the central *m* = −1/2 → *m* = +1/2 transition to measure *T*_M_ over a range of temperatures for different electron spin concentrations. In contrast to 1/*T*_1_, a strong dependence upon the concentration was observed for *T*_M_, suggesting that electron spin–spin coupling plays a significant role in driving decoherence.^26,27^ Panels c and d of Figure 3 show 1/*T*_M_ replotted as a function of the concentration at each temperature. At a given temperature, *T*_M_ was found to change linearly with the concentration and to obey the simple empirical relation
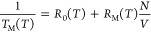
1where *R*_0_(*T*) is a concentration-independent rate and *R*_M_(*T*), which has units of μs^–1^ mM^–1^, characterizes the *T*_M_ concentration dependence.

**Figure 3:**
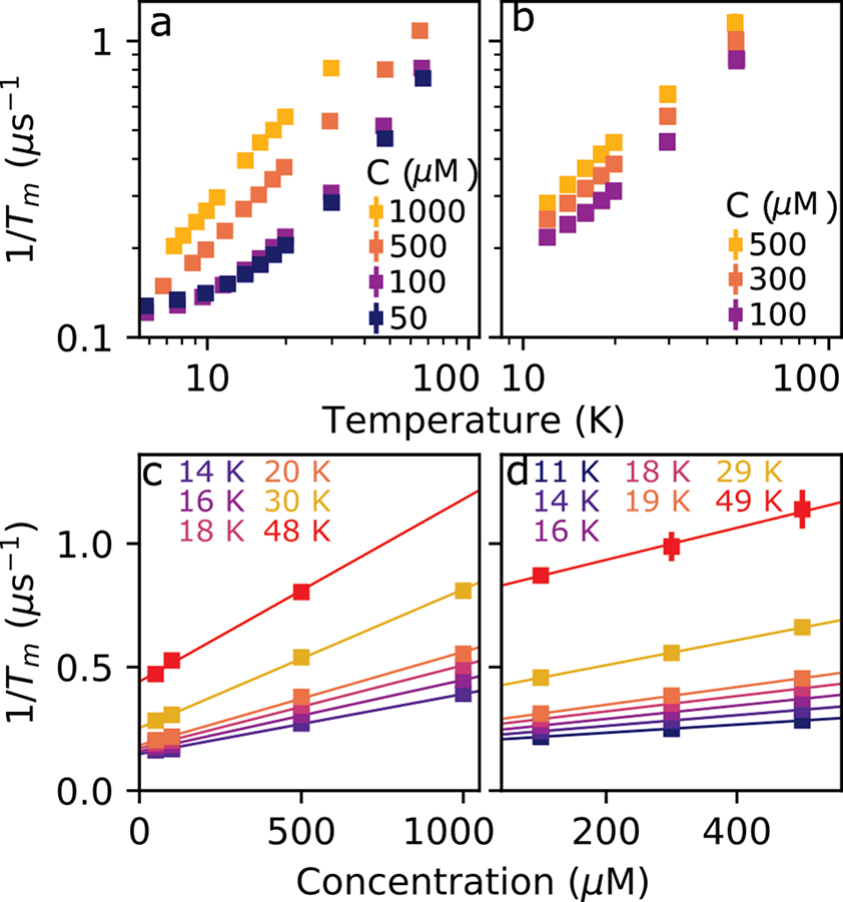
Inverse phase memory time 1/*T*_M_ of (a) Gd-DOTA and (b) Gd-PyMTA shows a strong temperature dependence. (c and d) 1/*T*_M_ plotted as a function of the concentration, at the indicated temperatures for (c) Gd-DOTA and (d) Gd-PyMTA. Solid lines indicate fits of 1/*T*_M_ to eq 1.

Coupling between the electron spins can be characterized by the average nearest-neighbor dipolar coupling strength ω_dd_(*r̅*), which is proportional to the electron spin concentration *N*/*V*^27,28^
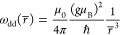
2that scales with the inverse cube of the average nearest-neighbor distance between electrons *r̅*, given by
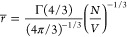
3where Γ is the Gamma function and Γ(4/3)/(4π/3)^−1/3^ ≃ 0.554.^29^Table 1 lists a range of electron spin concentrations and the corresponding average nearest-neighbor distances derived from eqs 2 and 3.

**Table 1:** Average Nearest-Neighbor Distances *r̅* and Electron–Electron Coupling Strengths ω_dd_(*r̅*) Assuming Randomly and Uniformly Distributed Spins in a Glassy Matrix for Several Spin Concentrations, Given by eq 3

concentration	*r̅* (nm)	ω_dd_(*r̅*)/2π (kHz)
1 mM	6.6	180
500 μM	8.3	91
100 μM	14.1	19
50 μM	17.8	9

To explain the observed dependence of *T*_M_ upon the temperature and electron spin concentration, we propose a model that explicitly includes both spin–lattice and spin–spin coupling. To account for the effects of spin–spin coupling, it is important to consider that all transitions except for the central *m* = −1/2 → *m* = +1/2 transition are significantly broadened by zero-field splitting. Therefore, most Gd^3+^ spins are not excited by microwave pulses. Only a small percentage of spins, termed “A” spins, are excited, while most spins, termed “B” spins, are left unexcited. Spin–spin interactions that lead to decoherence are overwhelmingly dominated by coupling between the rare, excited “A” spins and the much more abundant, unexcited “B” spins and the dynamics of the latter, which lead to fluctuations in the dipolar field seen by the “A” spins.

In our model of decoherence for the central *m* = −1/2 → *m* = +1/2 transition, we consider four principal decoherence mechanisms for the observed A spins: (1) direct *T*_1_ spin–lattice relaxation processes of the “A” spins, (2) coupling between “A” spins and nearby nuclear spins, and (3) fluctuations in the electron dipolar field seen by “A” spins driven by spin–lattice relaxation of “B” spins (“*T*_1_-induced” mechanism) or (4) energy-conserving pairwise “B” spin flip-flops that give rise to fluctuations in the spin bath (Figure 1). These four mechanisms, each with distinct physical origins, are discussed below.

Direct spin–lattice decoherence processes (mechanism 1), which occur at or near the electron Larmor frequency of the “A” spins, lead to direct “*T*_1_” relaxation and, therefore, coherence loss of “A” spins.^25^ These processes follow the temperature dependence of *T*_1_ (Figure 2) and are influenced by both direct spin–phonon coupling and zero-field splitting modulation.^30^ Because *T*_1_ and *T*_M_ differ by <10×, direct spin–lattice decoherence processes are expected to contribute heavily to decoherence.

Nuclear spins coupled to “A” spins (mechanism 2) drive decoherence through a different mechanism: pairs of nuclear spins can undergo spin flip-flops, where one nuclear spin flips from *m* → *m* + 1, while its neighbor flops from *m* + 1 → *m*, so that the total energy is conserved when the two nuclear spins have the same or very close Larmor frequencies. Nuclear spin flip-flops are driven by dipolar coupling between nuclear spins and lead to a time-varying magnetic field as seen by nearby “A” spins. Each nuclear spin pair produces a small fluctuation because their energy differences are tiny, but the effect of many spin pairs together produces a time-varying field that is large enough to lead to time-varying changes in electron spin precession, which are not refocused in a two-pulse Hahn echo, leading to a permanent loss of “A” spin phase coherence in a process known as nuclear spin-driven spectral diffusion.^31,32^ Careful treatment of the couplings between the nuclear spin bath and electron spins have shown that this is a partially coherent phenomenon.^33−35^ Except at ∼millikelvin temperatures, nuclear spin flip-flops in solids occur at a temperature-independent rate. Hence, at 8.6 T between 10 and 50 K, nuclear spin-driven spectral diffusion is temperature-independent.

The “*T*_1_-induced” mechanism (mechanism 3) describes a process in which a “B” spin near an “A” spin undergoes a spin flip as a result of its spin–lattice relaxation, leading to a change in the dipolar field seen by the “A” spin. Because electron–electron dipolar coupling is much stronger than electron–nuclear dipolar coupling, a single “B” spin flip is much more impactful than a single nuclear spin flip and can lead to a change in the precession of the “A” spin, which is not refocused in a two-pulse Hahn echo. This process is expected to drive “A” spin decoherence at a rate proportional to *T*_1_^–1^ and proportional to the strength of the average electron–electron dipolar coupling ω_dd_(*r̅*)^31,32^ (see Table 1). For typical spin-1/2 organic radicals, where *T*_1_ is much longer than *T*_M_, the “*T*_1_-induced” mechanism is not important.^27^ In contrast, for Gd^3+^, where *T*_1_ and *T*_M_ only differ by <10×, fast *T*_1_ can drive *T*_M_.

The fourth mechanism, energy-conserving pairwise electron spin flip-flops, is similar in some respects to pairwise nuclear spin flip-flops but is crucially different in character. Dipolar coupling can drive electron spin flip-flops if the dipolarly coupled spin pairs have the same or similar Larmor frequencies. Here, the electron flip-flop mechanism drives “A” electron spin dephasing through both an indirect process (Figure 1), where two neighboring “B” spins undergoing energy-conserving flip-flops that modify the precession of a nearby “A” spin, and a direct process, where a “B” spin and an “A” spin undergo mutual flip-flop. The changing dipolar field caused by the indirect flip-flop process leads to “A” spin decoherence through electron spin spectral diffusion,^31,32^ while the direct flip-flop process immediately destroys “A” spin coherence. Mutual flip-flops between pairs of “A” spins are extremely unlikely because most spins are not excited by microwave pulses applied to the central transition.

Another way in which electron-spin flip-flops differ from nuclear spin flip-flops is that electron spin flip-flops have a strong temperature dependence near the Zeeman temperature *T*_Z_ = *g*μ_B_*B*_0_/*k*_B_, which is 11.6 K at the high magnetic field of 8.6 T. At temperatures approaching *T*_Z_, flip-flops begin to “freeze out” as higher Zeeman levels become thermally depopulated.^27,28,36^ Below *T*_Z_, where the electron spins become completely polarized, there are no nearby pairs of electron spins that can undergo energy-conserving flip-flops. In high-spin systems, modeling the temperature dependence of electron spin flip-flops is challenging for a number of reasons, including that there are multiple transitions that depopulate at different temperatures and that lines are generally broad, so that two spins in the same Zeeman level may not be able to undergo energy-conserving flip-flops. A model for electron-spin flip-flops in crystalline high-spin systems was proposed by Takahashi et al., which predicts flip-flops to occur between neighboring spins at a rate *W* given by eq 4(26)
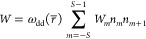
4where *n*_*m*_ is the Boltzmann population of Zeeman level *m* and *W*_*m*,*m*+1_ is the flip-flop matrix element
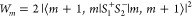
5which equates to *W*_*m*_ = 2((*S* – *m*)(*S* + *m* + 1))^2^.

The “crystalline flip-flop” model proposed by Takahashi et al. successfully described the low-temperature *T*_M_ dependence of an ensemble of crystallized *S* = 10 molecular magnets, which had large zero-field splittings and well-defined orientations, so that only transitions where one spin flips from *m* → *m* + 1 and another flips from *m* + 1 → *m* were energy-conserving (Figure 4c). Gd^3+^ complexes, on the other hand, have relatively small ZFS, with a broad distribution of ZFS values as a result of disorder.^16^ One important consequence is that, in a frozen glassy solution, EPR transitions between all Zeeman levels will overlap in frequency for some orientations and for some ZFS values,^16^ which is not conceptually accounted for in eq 4. As a result, flip-flops between any two Zeeman levels will be energy-conserving for some orientations and disorder realizations as long as one spin flips (*m* → *m* + 1) while the other flops (*m*′ → *m*′ – 1), as shown schematically in Figure 4d. We therefore propose a generalized high spin flip-flop model, where we explicitly consider energy-conserving flip-flops between all Zeeman transitions *m*,*m*′, where *m* → *m* + 1 as *m*′ → *m*′ – 1. Our proposed “ubiquitous flip-flop” model predicts the following contribution to electron spin decoherence
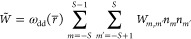
6where
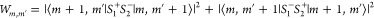
7Our full model for the coherence lifetime of the central *m* = −1/2 → *m* = +1/2 transition is given by
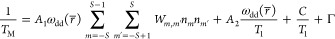
8where *A*_1_, *A*_2_, and *C* are concentration-independent factors and Γ is a residual relaxation rate. The first term reflects decoherence caused by energy-conserving electron spin flip-flops; the second term reflects decoherence driven by electron “*T*_1_-induced” neighboring spin flips; the third term reflects decoherence caused directly by the *T*_1_ processes of the “A” spins; and the fourth term is dominated by coupling to fluctuating nuclear spin flip-flops. The first two terms are proportional to the electron spin concentration because ω_dd_(*r̅*) ∝ *N*/*V* (eqs 2 and 3).

**Figure 4:**
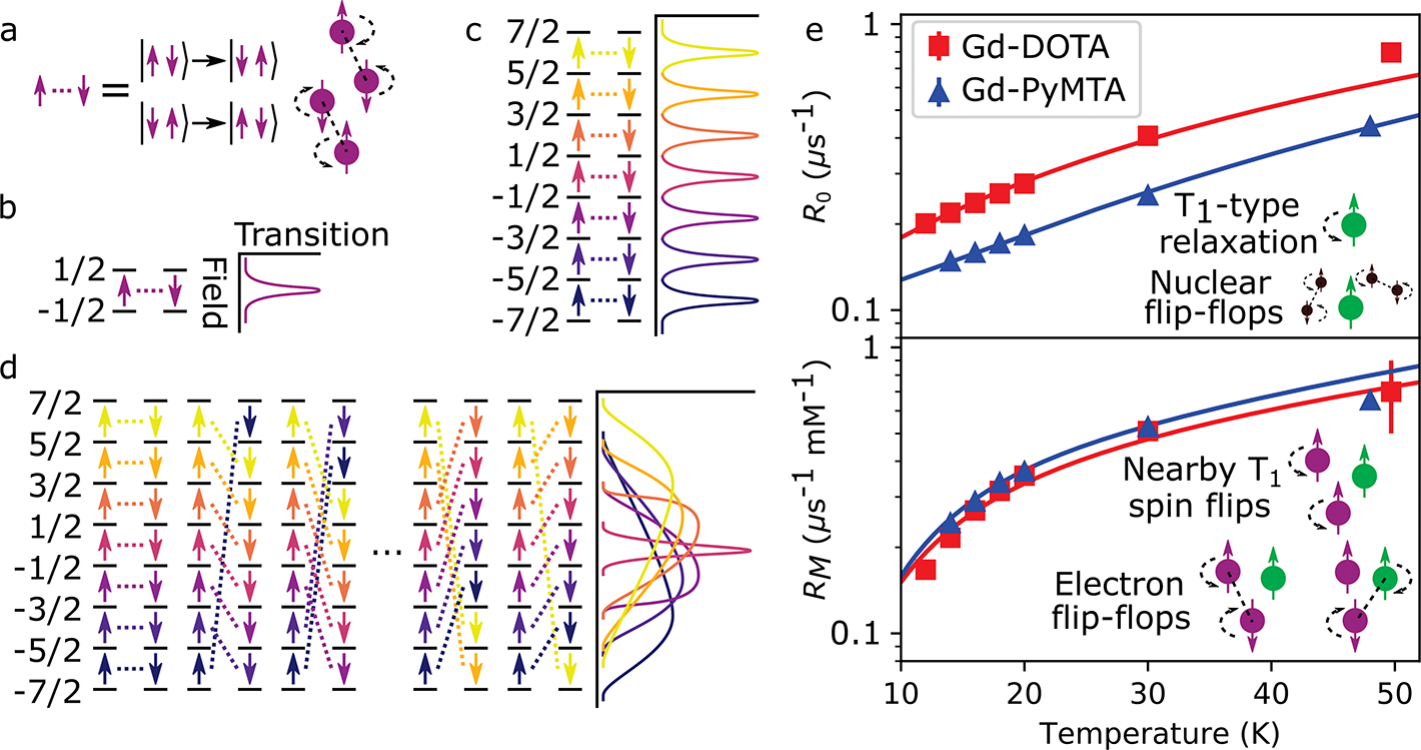
(a–d) Electron spin flip-flop models. (a) Energy-conserving flip-flop between a pair of spins driven by dipolar coupling. (b) Spin-1/2 flip-flop and the associated field-dependent EPR transition shown on the right. (c) Crystalline high-spin flip-flops for *S* = 7/2 and the associated EPR transitions. Large ZFS and small disorder ensure that EPR transitions are well-separated in energy, so that only *m* → *m* ± 1 flip-flops are energy-conserving.^26^ (d) Ubiquitous high-spin flip-flops for *S* = 7/2 and the associated EPR transitions. In an amorphous frozen solution of Gd centers, small ZFS, large disorder, and many orientations ensure that, at a particular magnetic field, energy-saving flip-flops can occur between all spin states for some orientations and disorder configurations. Field-dependent transitions are shown on the right. (e) Concentration-independent rate *R*_0_ (top) and concentration-dependent rate *R*_M_ (bottom) as a function of the temperature. Solid lines indicate fits to eq 9b, with parameters shown in Table 2. Insets show the relevant decoherence mechanisms.

Figure 4 shows a fit of our model for the coherence lifetime of the *m* = −1/2 → *m* = +1/2 transition to the observed decoherence rates extracted from eq 1. Following eq 8, the two empirical decoherence rates *R*_0_ and *R*_M_ were fit according to
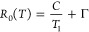
9a
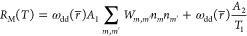
9bwhere *A*_1_, *A*_2_, *C*, and Γ were the four adjustable parameters. The results of the fitting procedure are presented in Table 2. *A*_1_ is roughly 50% larger for Gd-DOTA than for Gd-PyMTA, while *C* is roughly 50% larger for Gd-PyMTA, and *A*_2_ and Γ are the same for the two complexes.

**Table 2:** Model Parameters from Equation 8 Fit to the Temperature and Concentration Dependence of 1/*T*_M_ (Figure 4)

	Gd-DOTA	Gd-PyMTA
*A*_1_ (×10^–3^)	1.5 ± 0.1	0.9 ± 0.3
*A*_2_ (μs)	1.4 ± 0.2	1.4 ± 0.4
*C*	1.2 ± 0.1	1.8 ± 0.1
Γ (μs^–1^)	0.05 ± 0.01	0.05 ± 0.01
*r*_c_ (nm)	7.7 ± 0.3	7.7 ± 0.8

Equation 8 oversimplifies the electron flip-flop mechanism in two key ways. First, it considers only flip-flops between nearest neighbors, potentially underestimating the contribution of flip-flops to decoherence. Dipolar-coupled electron spins have many opportunities to undergo mutual electron spin flip-flops, including through couplings to nearby nuclear spins that make up for their energy differences in a three-spin electron–electron–nuclear spin process, also known as the cross effect.^37^ Second, it assumes all pairs of transitions Δ*m* = ±1 contribute equally to decoherence. Because each Δ*m* = ±1 transition is broadened by zero-field splitting to a different extent,^16^ not all pairs of transitions will overlap in frequency, and therefore, many pairs will not be energy-conserving. A full and accurate treatment of the flip-flop mechanism should take into account transition-dependent details of the EPR line shape. Equation 8 likely underestimates the contribution to flip-flops from the narrow central *m* = −1/2 → *m* = 1/2 transition, while overestimating the contributions from the other transitions, which are much broader. However, to a first approximation, we can expect the contribution of the flip-flop mechanism to dephasing to scale inversely with the full width of the EPR line, because the narrower the entire EPR line, including all transitions, the more likely neighboring spins will have transitions that occur at the same frequency. Taking zero-field splitting as a proxy for the full EPR line width, we note that the ZFS is half as large for Gd-DOTA as it is for Gd-PyMTA.^16^ This is consistent with our finding that *A*_1_, which scales the contribution from the flip-flop mechanism, is twice as large for Gd-DOTA as that for Gd-PyMTA.

The *S* = 1/2 transition of the nitroxide radical 4-amino-TEMPO at 8.6 T/240 GHz has a line width roughly 10–30 times narrower than the Gd^3+^ complexes investigated here.^16^ Accordingly, our model predicts a 10–30× larger *A*_1_ parameter for nitroxide radicals than for these Gd^3+^ complexes. Edwards et al. performed *T*_M_ measurements on frozen aqueous solutions of 4-amino-TEMPO at 8.6 T/240 GHz and found that *T*_M_ was well-modeled at low temperatures by , where Γ′ is a concentration- and temperature-independent rate,^27^ which recapitulates eq 8 for *S* = 1/2 with the terms proportional to *A*_2_ and *C* equal to 0, up to a factor of 2 in the definition of the term proportional to *A*_1_. We find the coefficient *A*_1_^4-amino-TEMPO^ = 1/10.2 × 1/2 = 0.049, which is indeed roughly ∼30 times larger than *A*_1_ for either Gd^3+^ complex measured.

In contrast, decoherence of “A” spins caused by *T*_1_-induced spin-flips of nearby “B” spins is not expected to depend upon details of the EPR line shape because it is driven by spin–lattice relaxation. This matches our finding that the *A*_2_ parameter is the same for both complexes. Rather, these spin-flips occur in “B” spins at a rate of 1/*T*_1_ and drive decoherence through dipolar coupling to “A” spins. The *A*_2_ parameter has units of time and scales the contribution to spin decoherence from the *T*_1_-induced spin-flip mechanism. A possible physical interpretation is that *A*_2_ = 1/ω_dd_(*r*_c_), where ω_dd_(*r*_c_) is the dipolar coupling frequency between two spins separated by a characteristic distance *r*_c_. If spins are much farther apart than *r*_c_, then their coherence lifetimes are not much affected by *T*_1_ flips of their neighbors. Our model gives characteristic distances *r*_c_ of 7.7 ± 0.3 and 7.7 ± 0.8 nm for Gd-DOTA and for Gd-PyMTA, respectively. Equation 8 can be re-expressed in terms of *r*_c_ as
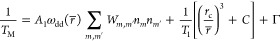
10with the contribution to decoherence from “*T*_1_-induced” “B” spin-flips falling off as (*r*_c_/*r̅*)^3^. Our model predicts that “A” spins are only strongly affected by *T*_1_-induced flips of “B” spins within a characteristic distance *r*_c_.

Decoherence driven by spin–lattice processes was weighted by the dimensionless parameter *C*. *T*_1_ processes are often mediated by the zero-field splitting, which is roughly twice as large in Gd-PyMTA as in Gd-DOTA, consistent with our finding that *C* was nearly twice as large for Gd-PyMTA as for Gd-DOTA. Residual relaxation Γ, independent of the temperature and concentration, is dominated by weak coupling between electron “A” spins and an ensemble of nuclear spins. Nuclear spins, which are not highly polarized at these temperatures, readily undergo energy-conserving flip-flops and cause the magnetic field seen by “A” spins to fluctuate.

Our quantitative model of electron spin decoherence can be used to extract average interelectron distances *r̅* from *T*_M_ and *T*_1_ measurements at several temperatures. Temperature-dependent *T*_M_ measurements of *S* = 1/2 nitroxide radicals have been shown to be sensitive to *r̅* as long as 6.6 nm.^27^ In our work, sensitivity to interelectron distances is shown for average interelectron distances of up to 17 nm (the average nearest interelectron distance for a 50 μM solution). Two crucial details provide this nearly 3× increase in maximum interspin distance sensitivity. First, the larger magnetic moment of *S* = 7/2 Gd^3+^ systems leads to stronger electron–electron coupling, as seen from the flip-flop matrix elements of eq 7. The matrix elements *W*_*m*,*m*′_ equate to *W*_*m*,*m*′_ = 2(*S* – *m*)(*S* + *m* + 1)(*S* + *m*′)(*S* – *m*′ + 1), which are 2–3 orders of magnitude larger for *S* = 7/2 than for *S* = 1/2. Second, *T*_1_ relaxation in Gd^3+^ spins is much shorter than that for nitroxide radicals, for which “*T*_1_-induced” spin-flips of “B” spins can effectively be ignored as a contribution to electron spin decoherence.^27^ The “*T*_1_-induced” mechanism provides an extra decoherence pathway for Gd^3+^ systems, leading to an increase in the sensitivity of *T*_M_ measurements to electron–electron coupling. Measurements of average interelectron distances using dephasing and relaxation-based techniques have great potential as tools for understanding molecular aggregation and clustering in frozen solutions, especially when aggregation is driven by weak interactions and intermolecular associations are not strong. Moreover, such measurements are sensitive to the geometrical arrangement of spins,^27^ providing important additional and complementary information to pulsed dipolar spectroscopy techniques, which are most sensitive to pairwise distances.

The measurements and model that we have presented advance our quantitative understanding of spin relaxation and decoherence of half-integer high-spin paramagnetic centers, like Gd^3+^, in high magnetic fields and at relevant concentrations used for biophysics, structural biology, quantum sensing, and MRI applications. Our quantitative model of decoherence for Gd^3+^ complexes can inform the design of materials and experiments for which controlling decoherence is important. For example, for pulsed dipolar spectroscopy using Gd^3+^ spin labels, the model proposed here could be used to optimize the temperature, spin label concentration, and nuclear spin concentration (by, for example, deuteration) for measurements of pairwise distances in spin-labeled biological molecules and materials. A similar model may also be useful to understand decoherence of molecular qubits based on high-spin paramagnetic centers.^10,11,38^

## Supporting Information Available

Experimental methods, field-swept echo-detected EPR line shapes, relaxation data details, and comparing crystalline and ubiquitous flip-flop models (PDF)

## References

[ref1] Advanced ESR Methods in Polymer Research; Schlick S., Ed.; Wiley-Interscience: Hoboken, NJ, 2006; 10.1002/047005350X.

[ref2] Multifrequency Electron Paramagnetic Resonance Theory and Applications; Misra S. K., Ed.; Wiley-VCH: Weinheim, Germany, 2011; 10.1002/9783527633531.

[ref3] Van Doorslaer S. Hyperfine Spectroscopy: ESEEM. eMagRes. 2017, 6, 51–70. 10.1002/9780470034590.emrstm1517.

[ref4] Cornish V. W.; Benson D. R.; Altenbach C. A.; Hideg K.; Hubbell W. L.; Schultz P. G. Site-Specific Iincorporation of Biophysical Probes Into Proteins. Proc. Natl. Acad. Sci. U. S. A. 1994, 91, 2910–2914. 10.1073/pnas.91.8.2910. 8159678 PMC521698

[ref5] Hussain S.; Franck J. M.; Han S. Transmembrane Protein Activation Refined by Site-Specific Hydration Dynamics. Angew. Chem., Int. Ed. 2013, 52, 1953–1958. 10.1002/anie.201206147. 23307344

[ref6] Jeschke G. DEER Distance Measurements on Proteins. Annu. Rev. Phys. Chem. 2012, 63, 419–446. 10.1146/annurev-physchem-032511-143716. 22404592

[ref7] Zadrozny J. M.; Niklas J.; Poluektov O. G.; Freedman D. E. Millisecond Coherence Time in a Tunable Molecular Electronic Spin Qubit. ACS Central Science 2015, 1, 488–492. 10.1021/acscentsci.5b00338. 27163013 PMC4827467

[ref8] Bonizzoni C.; Ghirri A.; Atzori M.; Sorace L.; Sessoli R.; Affronte M. Coherent Coupling between Vanadyl Phthalocyanine Spin Eensemble and Microwave Photons: Towards Integration of Molecular Spin Qubits into Quantum Circuits. Sci. Rep. 2017, 7, 13096 10.1038/s41598-017-13271-w. 29026118 PMC5638858

[ref9] Atzori M.; Sessoli R. The Second Quantum Revolution: Role and Challenges of Molecular Chemistry. J. Am. Chem. Soc. 2019, 141, 11339–11352. 10.1021/jacs.9b00984. 31287678

[ref10] Zhou S.; Yuan J.; Wang Z.-Y.; Ling K.; Fu P.-X.; Fang Y.-H.; Wang Y.-X.; Liu Z.; Porfyrakis K.; Briggs G. A. D.; Gao S.; Jiang S.-D. Implementation of Quantum Level Addressability and Geometric Phase Manipulation in Aligned Endohedral Fullerene Qudits. Angew. Chem., Int. Ed. 2022, 61, e202115263 10.1002/anie.202115263. 34913233

[ref11] Fu P.-X.; Zhou S.; Liu Z.; Wu C.-H.; Fang Y.-H.; Wu Z.-R.; Tao X.-Q.; Yuan J.-Y.; Wang Y.-X.; Gao S.; Jiang S.-D. Multiprocessing Quantum Computing through Hyperfine Couplings in Endohedral Fullerene Derivatives. Angew. Chem., Int. Ed. 2022, 61, e202212939 10.1002/anie.202212939. 36310119

[ref12] Raitsimring A. M.; Gunanathan C.; Potapov A.; Efremenko I.; Martin J. M. L.; Milstein D.; Goldfarb D. Gd^3+^ Complexes as Potential Spin Labels for High Field Pulsed EPR Distance Measurements. J. Am. Chem. Soc. 2007, 129, 14138–14139. 10.1021/ja075544g. 17963387

[ref13] Potapov A.; Yagi H.; Huber T.; Jergic S.; Dixon N. E.; Otting G.; Goldfarb D. Nanometer-Scale Distance Measurements in Proteins Using Gd^3+^ Spin Labeling. J. Am. Chem. Soc. 2010, 132, 9040–9048. 10.1021/ja1015662. 20536233

[ref14] Edwards D.; Huber T.; Hussain S.; Stone K.; Kinnebrew M.; Kaminker I.; Matalon E.; Sherwin M.; Goldfarb D.; Han S. Determining the Oligomeric Structure of Proteorhodopsin by Gd^3+^-Based Pulsed Dipolar Spectroscopy of Multiple Distances. Structure 2014, 22, 1677–1686. 10.1016/j.str.2014.09.008. 25438671

[ref15] Razzaghi S.; Qi M.; Nalepa A. I.; Godt A.; Jeschke G.; Savitsky A.; Yulikov M. RIDME Spectroscopy with Gd(III) Centers. J. Phys. Chem. Lett. 2014, 5, 3970–3975. 10.1021/jz502129t. 26276479

[ref16] Clayton J. A.; Keller K.; Qi M.; Wegner J.; Koch V.; Hintz H.; Godt A.; Han S.; Jeschke G.; Sherwin M. S.; Yulikov M. Quantitative Analysis of Zero-Field Splitting Parameter Distributions in Gd(III) Complexes. Phys. Chem. Chem. Phys. 2018, 20, 10470–10492. 10.1039/C7CP08507A. 29617015 PMC6026474

[ref17] Seal M.; Zhu W.; Dalaloyan A.; Feintuch A.; Bogdanov A.; Frydman V.; Su X.-C.; Gronenborn A. M.; Goldfarb D. Gd^III^-^19^F Distance Measurements for Proteins in Cells by Electron-Nuclear Double Resonance. Angew. Chem., Int. Ed. 2023, 62, e202218780 10.1002/anie.202218780. 36905181

[ref18] Clayton J. A.; Qi M.; Godt A.; Goldfarb D.; Han S.; Sherwin M. S. Gd^3+^–Gd^3+^ Distances Exceeding 3 nm Determined by Very High Frequency Continuous Wave Electron Paramagnetic Resonance. Phys. Chem. Chem. Phys. 2017, 19, 5127–5136. 10.1039/C6CP07119H. 28139788 PMC5394103

[ref19] Jeschke G.; Polyhach Y. Distance Measurements on Spin-Labelled Biomacromolecules by Pulsed Electron Paramagnetic Resonance. Phys. Chem. Chem. Phys. 2007, 9, 1895–1910. 10.1039/b614920k. 17431518

[ref20] Cohen M. R.; Frydman V.; Milko P.; Iron M. A.; Abdelkader E. H.; Lee M. D.; Swarbrick J. D.; Raitsimring A.; Otting G.; Graham B.; Feintuch A.; Goldfarb D. Overcoming Artificial Broadening in Gd^3+^–Gd^3+^ Distance Distributions Arising from Dipolar Pseudo-Secular Terms in DEER Experiments. Phys. Chem. Chem. Phys. 2016, 18, 12847–12859. 10.1039/C6CP00829A. 27102158

[ref21] Manukovsky N.; Feintuch A.; Kuprov I.; Goldfarb D. Time Domain Simulation of Gd^3+^–Gd^3+^ Distance Measurements by EPR. J. Chem. Phys. 2017, 147, 044201 10.1063/1.4994084. 28764344

[ref22] Keller K.; Mertens V.; Qi M.; Nalepa A. I.; Godt A.; Savitsky A.; Jeschke G.; Yulikov M. Computing Distance Distributions from Dipolar Evolution Data with Overtones: RIDME Spectroscopy with Gd(III)-Based Spin Labels. Phys. Chem. Chem. Phys. 2017, 19, 17856–17876. 10.1039/C7CP01524K. 28660955

[ref23] Takahashi S.; Brunel L.-C.; Edwards D. T.; van Tol J.; Ramian G.; Han S.; Sherwin M. S. Pulsed Electron Paramagnetic Resonance Spectroscopy Powered by a Free-Electron Laser. Nature 2012, 489, 409–13. 10.1038/nature11437. 22996555

[ref24] Edwards D. T.; Ma Z.; Meade T. J.; Goldfarb D.; Han S.; Sherwin M. S. Extending the Distance Range Accessed with Continuous Wave EPR with Gd^3+^ Spin Probes at High Magnetic Fields. Phys. Chem. Chem. Phys. 2013, 15, 11313–11326. 10.1039/c3cp43787f. 23732863 PMC4142211

[ref25] Schweiger A.; Jeschke G. Principles of Pulse Electron Paramagnetic Resonance; Oxford University Press: Oxford, U.K., 2001.

[ref26] Takahashi S.; van Tol J.; Beedle C. C.; Hendrickson D. N.; Brunel L.-C.; Sherwin M. S. Coherent Manipulation and Decoherence of *S* = 10 Single-Molecule Magnets. Phys. Rev. Lett. 2009, 102, 087603 10.1103/PhysRevLett.102.087603. 19257788

[ref27] Edwards D. T.; Takahashi S.; Sherwin M. S.; Han S. Distance Measurements Across Randomly Distributed Nitroxide Probes from the Temperature Dependence of the Electron Spin Phase Memory Time at 240 GHz. J. Magn. Reson. 2012, 223, 198–206. 10.1016/j.jmr.2012.07.004. 22975249

[ref28] Takahashi S.; Hanson R.; van Tol J.; Sherwin M. S.; Awschalom D. D. Quenching Spin Decoherence in Diamond through Spin Bath Polarization. Phys. Rev. Lett. 2008, 101, 047601 10.1103/PhysRevLett.101.047601. 18764365

[ref29] Chandrasekhar S. Stochastic Problems in Physics and Astronomy. Rev. Mod. Phys. 1943, 15, 1–89. 10.1103/RevModPhys.15.1.

[ref30] Raitsimring A.; Dalaloyan A.; Collauto A.; Feintuch A.; Meade T.; Goldfarb D. Zero Field Splitting Fluctuations Induced Phase Relaxation of Gd^3+^ in Frozen Solutions at Cryogenic Temperatures. J. Magn. Reson. 2014, 248, 71–80. 10.1016/j.jmr.2014.09.012. 25442776 PMC4495766

[ref31] Klauder J. R.; Anderson P. W. Spectral Diffusion Decay in Spin Resonance Experiments. Phys. Rev. 1962, 125, 912–932. 10.1103/PhysRev.125.912.

[ref32] Salikhov K. M.; Dzuba S. A.; Raitsimring A. M. The Theory of Electron Spin-Echo Signal Eecay Resulting from Dipole–Dipole Interactions Between Paramagnetic Centers in Solids. J. Magn. Reson. 1981, 42 (2), 255–276. 10.1016/0022-2364(81)90216-X.

[ref33] Canarie E. R.; Jahn S. M.; Stoll S. Quantitative Structure-Based Prediction of Electron Spin Decoherence in Organic Radicals. J. Phys. Chem. Lett. 2020, 11, 3396–3400. 10.1021/acs.jpclett.0c00768. 32282218 PMC7654569

[ref34] Bahrenberg T.; Jahn S. M.; Feintuch A.; Stoll S.; Goldfarb D. The Decay of the Refocused Hahn Echo in Double Electron-Electron Resonance (DEER) Experiments. Magn. Reson. 2021, 2, 161–173. 10.5194/mr-2-161-2021. PMC10539729 37904783

[ref35] Jahn S. M.; Canarie E. R.; Stoll S. Mechanism of Electron Spin Decoherence in a Partially Deuterated Glassy Matrix. J. Phys. Chem. Lett. 2022, 13, 5474–5479. 10.1021/acs.jpclett.2c00939. 35687401 PMC9503049

[ref36] Kutter C.; Moll H. P.; van Tol J.; Zuckermann H.; Maan J. C.; Wyder P. Electron-Spin Echoes at 604 GHz Using Far Infrared Lasers. Phys. Rev. Lett. 1995, 74, 2925–2928. 10.1103/PhysRevLett.74.2925. 10058059

[ref37] Hovav Y.; Feintuch A.; Vega S. Theoretical Aspects of Dynamic Nuclear Polarization in the Solid State—The Cross Effect. J. Magn. Reson. 2012, 214, 29–41. 10.1016/j.jmr.2011.09.047. 22119645

[ref38] Hu Z.; Dong B.-W.; Liu Z.; Liu J.-J.; Su J.; Yu C.; Xiong J.; Shi D.-E.; Wang Y.; Wang B.-W.; Ardavan A.; Shi Z.; Jiang S.-D.; Gao S. Endohedral Metallofullerene as Molecular High Spin Qubit: Diverse Rabi Cycles in Gd_2_@C_79_N. J. Am. Chem. Soc. 2018, 140 (3), 1123–1130. 10.1021/jacs.7b12170. 29272584

