# Supplementary material for: Gadolinium Spin Decoherence Mechanisms at High Magnetic Fields
Source: J Phys Chem Lett. 2023 Nov 17;14(47):10578–84. doi: 10.1021/acs.jpclett.3c01847 (PMC10694812; doi:10.1021/acs.jpclett.3c01847)
Supplement: Supplementary file 1 — jz3c01847_si_001.pdf [file jz3c01847_si_001.pdf]

Supporting Information: Gadolinium Spin Decoherence Mechanisms at High Magnetic Fields

C. Blake Wilson,† Mian Qi,‡ Songi Han,¶,§,∥ and Mark S. Sherwin*,⊥,∥

†*Laboratory of Chemical Physics, National Institute of Diabetes and Digestive and Kidney Diseases, National Institutes of Health, Bethesda, Maryland, 20892, USA*

‡*Faculty of Chemistry and Center for Molecular Materials, Bielefeld University, 33615 Bielefeld, Germany*

¶*Department of Chemistry and Biochemistry, University of California, Santa Barbara, Santa Barbara, California, 93106, USA*

§*Department of Chemical Engineering, University of California, Santa Barbara, Santa Barbara, California, 93106, USA*

∥*Institute for Terahertz Science and Technology, University of California, Santa Barbara, Santa Barbara, California, 93106, USA*

⊥*Department of Physics, University of California, Santa Barbara, Santa Barbara, California, 93106, USA*

E-mail: sherwin@physics.ucsb.edu

Experimental details

Samples of Gd^{3+} chelates were dissolved in 60 % deuterated glycerol, 40 % D_2O (volume percent). For EPR measurements, 8 μl of sample were loaded into a Teflon sample holder.

Experiments were carried out using a home-built EPR spectrometer described previously[S1,S2] which operates at 240 GHz/8.6 T. Temperature control was achieved using a continuous flow cryostat (Janis Research Company) mounted inside the room-temperature bore of a superconducting magnet (Oxford Instruments). Cold helium gas was used for cooling. Microwaves were generated by a 55 mW solid-state source (Virginia Diodes, Inc.).

Figures S2 and S3 show field-swept echo-detected EPR spectra acquired around the central $m = -1/2 \rightarrow m = 1/2$ transition for Gd-DOTA and Gd-PyMTA, respectively. Spectra were acquired using a two-pulse echo of the form $P_1 - \tau - P_2 - \tau-$echo, where P_1, τ, P_2 were kept fixed and the echo was recorded as a function of magnetic field. Field sweeps were centered at 8608 T. Only the $m = -1/2 \rightarrow m = 1/2$ transition contributes to the peak shown at the center of the field sweep. All other transitions form a broad baseline which covers ± 0.4.[S3] The echo intensity outside of the central $m = -1/2 \rightarrow m = 1/2$ transition was at most $<5\%$ of the echo intensity at the peak of the central transition.

T_M measurements were carried out at the peak of the central $m = -1/2 \rightarrow m = 1/2$ transition using a two-pulse echo of the form $P_1 - \tau - P_2 - \tau-$echo. The integrated echo magnitude squared was recorded as a function of inter-pulse delay τ. Representative echo decay curves are shown in Figures S4 and S5. The phase memory time T_M was extracted by fitting the square of the echo magnitude $E(2\tau)^2$ with a function of the form $E(2\tau)^2 = A\exp(-2\tau/2T_M) + C$. Echo decays were observed to be well-described by a single exponential. A repetition period of 800 μs was used.

T_M experiments performed on Gd-DOTA were carried out with pulses $P_1 = 175$ ns and $P_2 = 275$ ns. Echo decay experiments were also performed with longer pulses $P_1 = 300$ ns and $P_2 = 510$ ns to confirm that instantaneous spectral diffusion was not significantly contributing to echo decay (Figure S4). T_M experiments performed on Gd-PyMTA were carried out using pulses $P_1 = P_2 = 350$ ns (Figure S5).

Figure S1 shows T_M plotted as a function of temperature (data from Figure 3 in the main text), along with fits to the full model of electron spin decoherence (Equation 10 in the main

text), using the fitting parameters shown in Table 2.

T_1 measurements were carried out using an echo-detected saturation-recovery experiment with a pulse sequence of the form $P_{\text{sat}} - T - P_1 - \tau - P_2 - \tau-$ echo, with saturation pulse $P_{\text{sat}} = 300$ μs. Representative datasets are shown in Figure S6. For Gd-DOTA, pulses $P_1 = 175$ ns, and $P_2 = 275$ ns were used. For Gd-PyMTA, pulses $P_1 = P_2 = 350$ ns were used. τ was kept fixed and was between 0.9 μs and 1.2 μs. The integrated echo was recorded as a function of the recovery period T. T_1 was extracted by fitting the integrated echo $E(T)$ with a function of the form $E(T) = A\exp(-T/T_1) + C$ (Figure S6.

Comparing flip-flop models

Figure S7 compares the temperature dependence of the flip-flop-driven decoherence term using either the crystalline model or the ubiquitous flip-flop models for S=7/2. The crystalline flip-flop model predicts a sharper initial temperature dependence, which flattens out at around 20K, while the ubiquitous flip-flop model predicts the temperature-dependent flip-flop rate continues to increase with temperature through 50 K.

The crystalline flip-flop model predicts the concentration-dependent decoherence rate R_M to be

$$R_M = \omega_{dd}(\bar{r})A_1 \sum_m W_m n_m n_{m+1} + \omega_{dd}(\bar{r})\frac{A_2}{T_1} \tag{1}$$

Figure S8 shows the temperature-dependent flip-flop rate R_M for Gd-DOTA and Gd-PyMTA (from Figure 4e in the main text), along with fits to the temperature dependence predicted by Equations 10 (using the ubiquitous flip-flop model) and 1 (using the crystalline flip-flop model. Fitting parameters for both models are shown in Table SS1. We note that from a conceptual standpoint, the crystalline model underestimates the prevalence of energy-conserving electron spin flip-flops taking place at nearly every temperature, since frozen Gd^{3+} centers are randomly oriented and display a wide distribution of zero field splitting parameters.[S3]

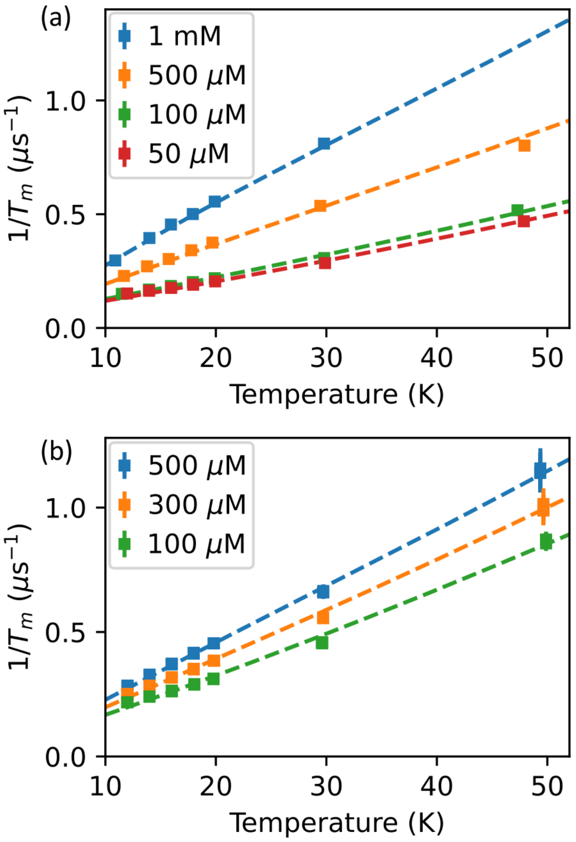

Figure S1: Inverse phase memory time $1/T_M$ of (a) Gd-DOTA and (b) Gd-PyMTA. Dashed lines represent a fit to Equation 10, the full model of $1/T_M$ concentration and temperature dependence.

Table S1: Model parameters extracted from fits fo the concentration-dependent decoherence rate R_M to Equation 10, using the ubiquitous flip-flop model, and to Equation S1, using the crystalline flip-flop model.

	Ubiquitous flip-flop model		Crystalline flip-flop model	
	Gd-DOTA	Gd-PyMTA	Gd-DOTA	Gd-PyMTA
$A1/10^{-}3$	1.5 ± 0.1	0.9 ± 0.3	3.2 ± 0.2	1.2 ± 0.6
A1 (μs)	1.4 ± 0.2	1.4 ± 0.4	2.9 ± 0.2	2.3 ± 0.3

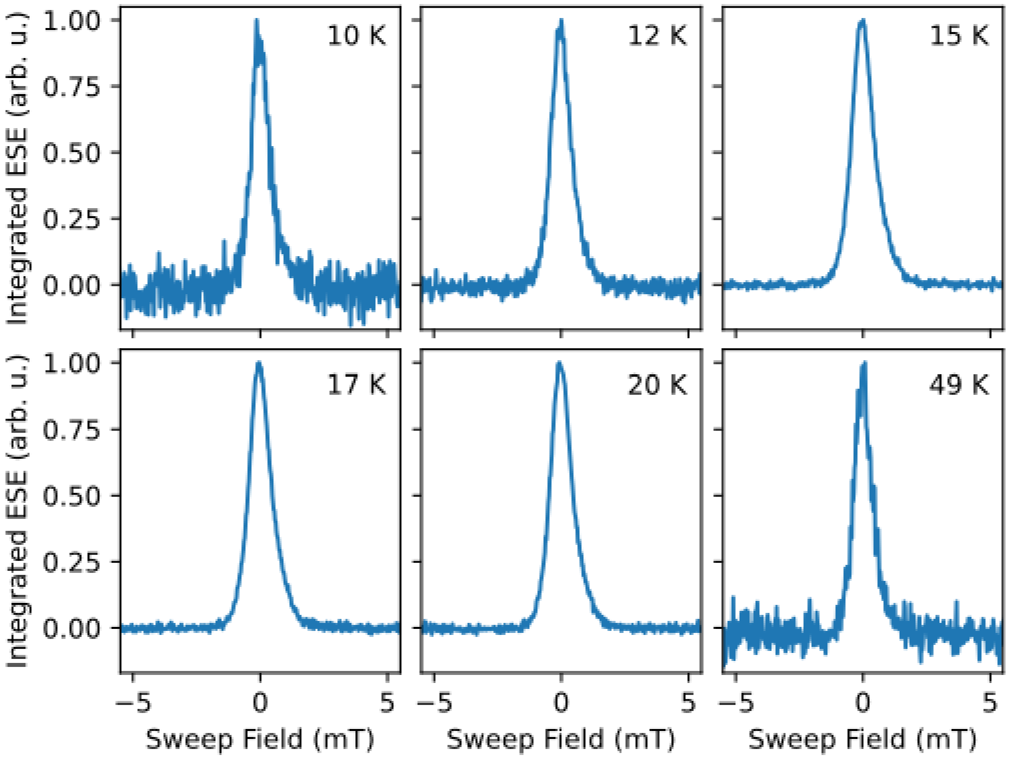

Figure S2: Field-swept echo-detected EPR spectra acquired on Gd-DOTA as a function of temperature, using a pulse sequence of the form $P_1 - \tau - P_2 - \tau -$ echo, where $P_1 = 175$ ns, $P_2 = 275$ ns, and $\tau = 900$ ns. Field sweeps were centered at 8608 T.

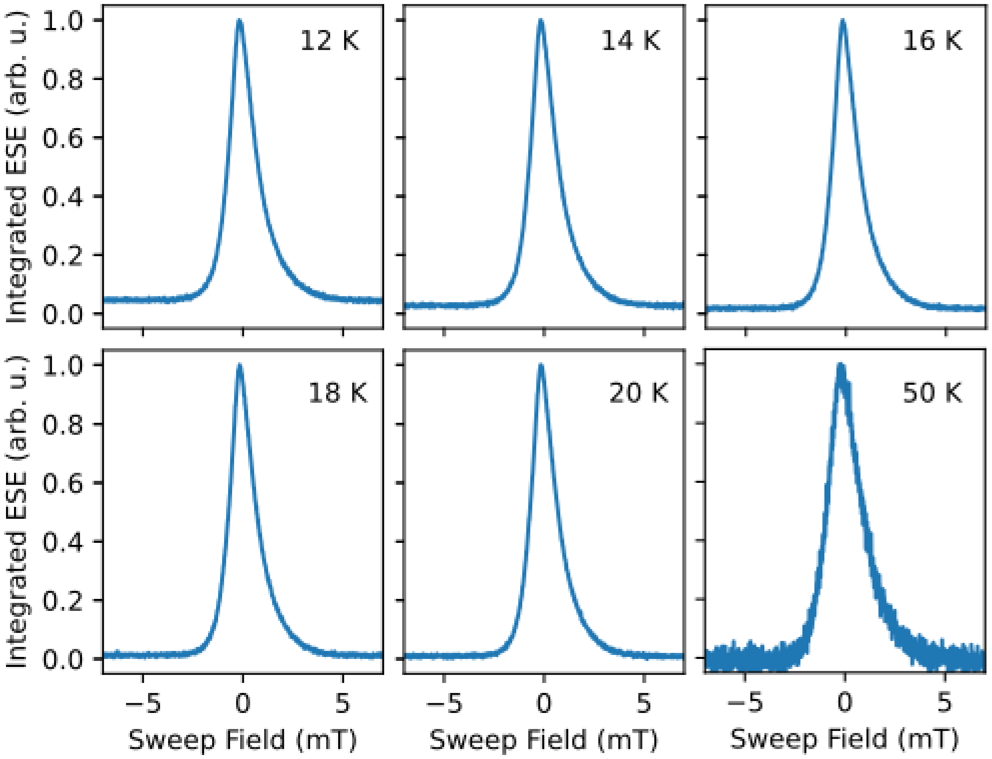

Figure S3: Field-swept echo-detected EPR spectra acquired on Gd-PyMTA as a function of temperature, using a pulse sequence of the form $P_1 - \tau - P_2 - \tau -$ echo, where $P_1 = P_2 =$ 350 ns, and $\tau = 1.0\ \mu$s. Field sweeps were centered at 8608 T.

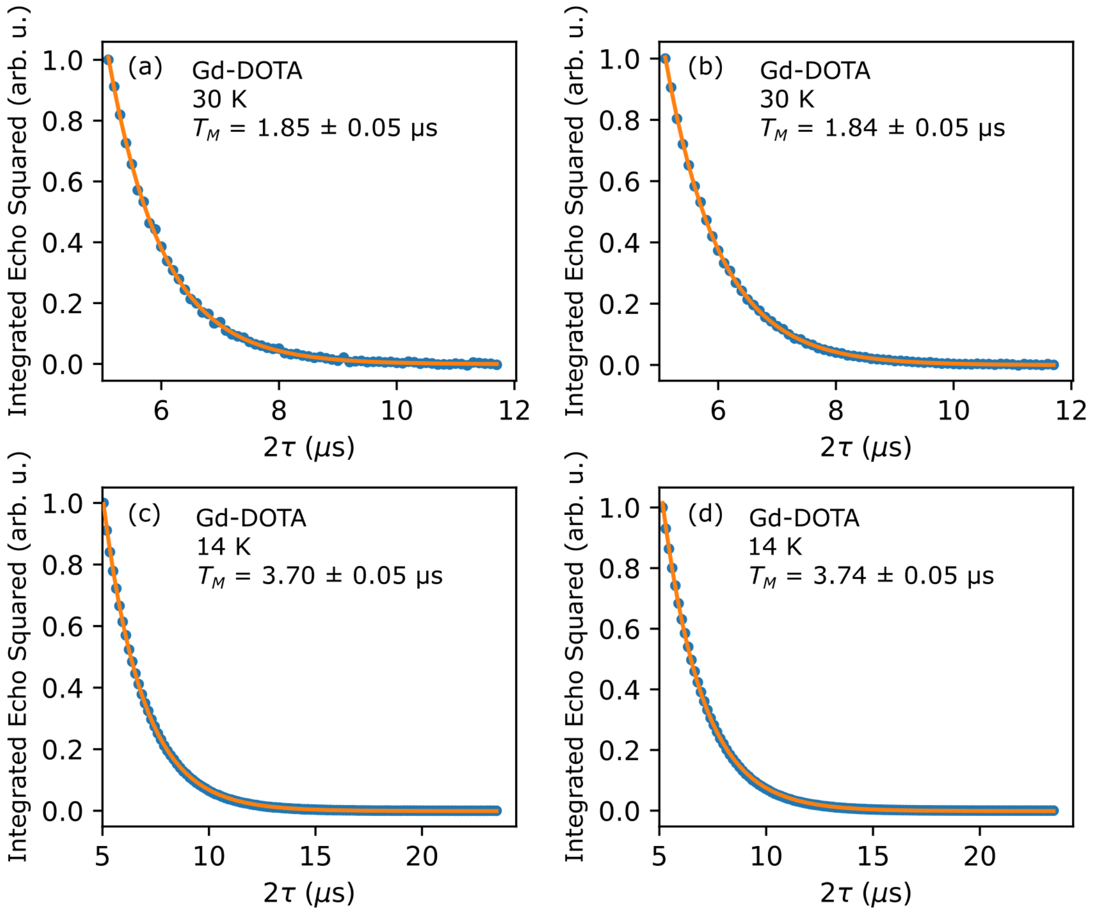

Figure S4: T_M experiments performed on Gd-DOTA at 30 K (a),(b) or 14 K (c),(d) using a pulse sequence of the form $P_1 - \tau - P_2 - \tau -$echo, using either pulse lengths $P_1 = 175$ ns, $P_2 = 275$ ns (a),(c) or $P_1 = 300$ ns, $P_2 = 510$ ns (b),(d). The integrated echo magnitude squared was recorded as a function of inter-pulse delay τ.

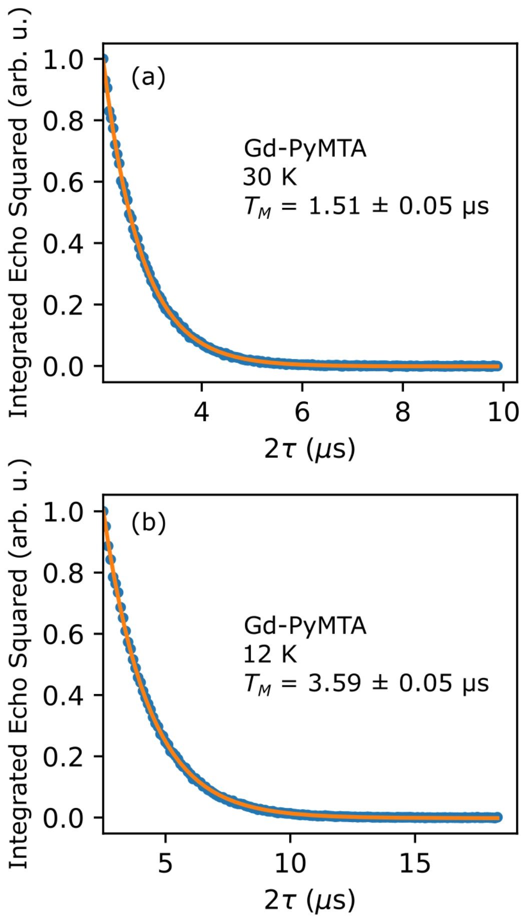

Figure S5: Electron spin-echo decay experiments carried out with a pulse sequence of the form $P_1 - \tau - P_2 - \tau -$echo, where $P_1 = P_2 = 350$ ns. The integrated echo magnitude squared was recorded as a function of inter-pulse delay τ.

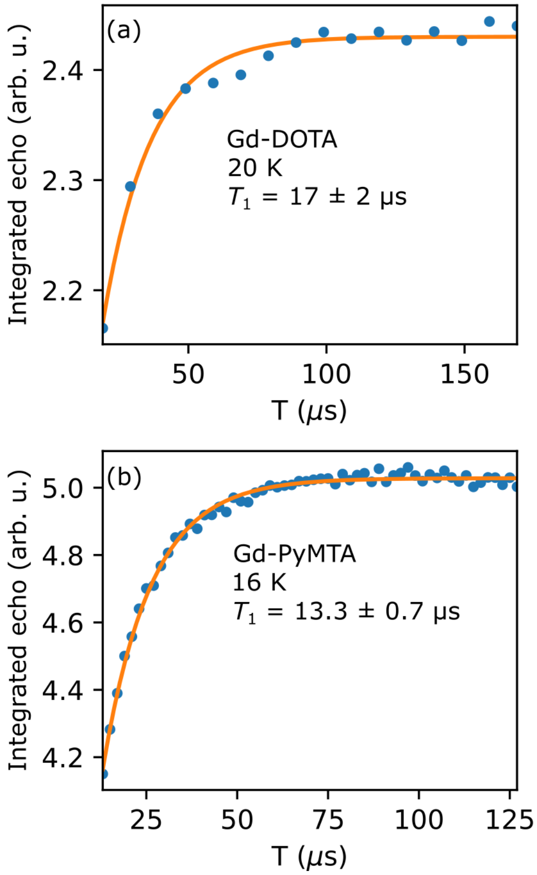

Figure S6: Saturation-recovery experiments were carried out using a pulse sequence of the form $P_{sat} - T - P_1 - \tau - P_2 - \tau -$ echo on Gd-DOTA (a), using $P_1 = 175$ ns, $P_2 = 275$ ns, and on Gd-PyMTA (b) using $P_1 = P_2 = 350$ ns. τ was kept fixed.

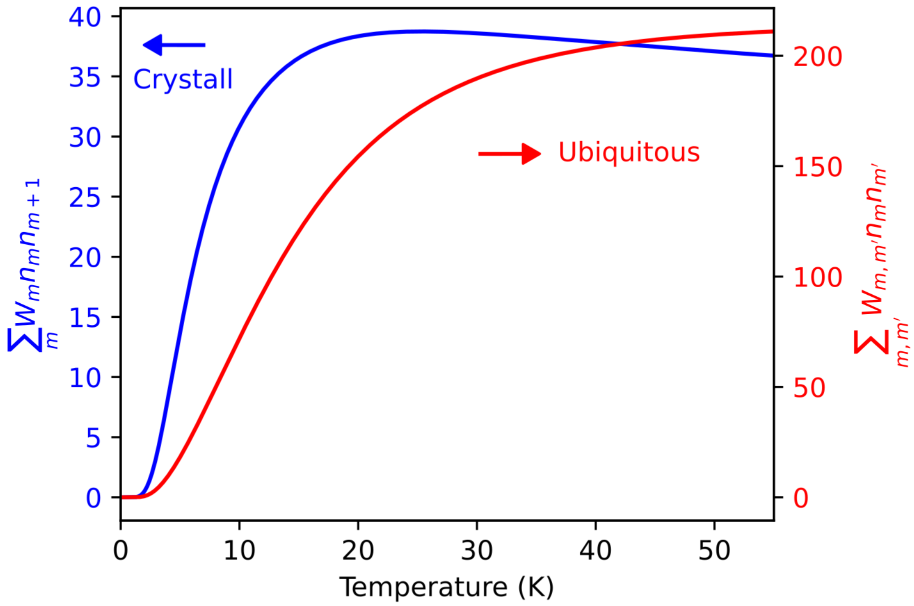

Figure S7: Temperature dependence of electron spin flip-flop-driven decoherence as predicted by the crystalline (Equation 4) and ubiquitous (Equation 6) models.

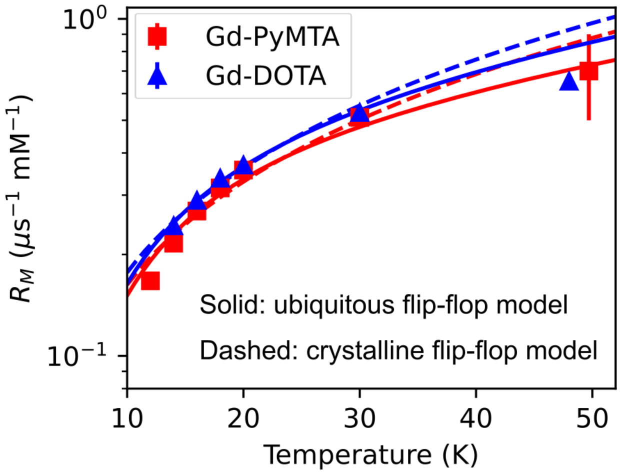

Figure S8: Concentration-dependent rate R_M plotted as a function of temperature, from Figure 4e. Solid lines indicate fits to Equation 9 using the ubiquitous flip-flop model. Dashed lines indicate a fit to Equation 9 using the crystalline flip-flop model, using the fitting parameters shown in Table S1.

References

(S1) Takahashi, S.; Brunel, L.-C.; Edwards, D. T.; van Tol, J.; Ramian, G.; Han, S.; Sherwin, M. S. Pulsed Electron Paramagnetic Resonance Spectroscopy Powered by a Free-Electron Laser. *Nature* **2012**, *489*, 409–13.

(S2) Edwards, D. T.; Takahashi, S.; Sherwin, M. S.; Han, S. Distance Measurements Across Randomly Distributed Nitroxide Probes from the Temperature Dependence of the Electron Spin Phase Memory Time at 240 GHz. *Journal of Magnetic Resonance* **2012**, *223*, 198 – 206.

(S3) Clayton, J. A.; Keller, K.; Qi, M.; Wegner, J.; Koch, V.; Hintz, H.; Godt, A.; Han, S.; Jeschke, G.; Sherwin, M. S.; Yulikov, M. Quantitative Analysis of Zero-Field Splitting Parameter Distributions in Gd(iii) Complexes. *Phys. Chem. Chem. Phys.* **2018**, *20*, 10470–10492.
